# Application evaluation of mixed-reality holographic imaging technology in the surgical treatment of spinal cord glioma

**DOI:** 10.12669/pjms.38.7.4777

**Published:** 2022

**Authors:** Zhenjie Liu, Xin Li, Jing Lu

**Affiliations:** 1 Zhenjie Liu, Department of Neurosurgery, Baoding No.1 Central Hospital, Baoding 071000, Hebei, China; 2 Xin Li, Department of Neurosurgery, Baoding No.1 Central Hospital, Baoding 071000, Hebei, China; 3 Jing Lu, Department of Oncology, Baoding Hengxing Integrated Chinese and Western Medicine, Hospital, Baoding 071000, Hebei, China

**Keywords:** Mixed-reality, Holographic imaging, Spinal cord glioma, Neurosurgery

## Abstract

**Objectives:**

To investigate the application effect of mixed reality (MR) holographic imaging technology in the clinical surgical treatment of spinal cord glioma.

**Methods:**

The clinical data of 53 patients with spinal cord glioma who underwent surgical treatment in the Neurosurgery Department of our hospital from January 2017 to May 2020 were retrospectively studied. All the patients were divided into two groups according to different assistive technologies during the surgery: the observation group and the control group, with 30 cases and 23 cases respectively. Patients in the observation group received MR holographic imaging technology intraoperatively, while those in the control group received ultrasound. The surgical conditions of the two groups: the rate of complete resection of tumor lesions and the evaluation accuracy of complete resection were compared. Patients were followed up for 12 months in the outpatient department after surgery, and the recovery of postoperative spinal physiological function was evaluated based on imaging review and MMS scale grading, and the recurrence was obtained.

**Results:**

There was no statistical significance in the basic clinical conditions between the two groups (P>0.05), and the total tumor resection rate in the experimental group was 96.67%, and that in the control group was 82.61%, showing a statistically significant difference (P<0.05). Based on enhanced MRI examination as the standard, the evaluation accuracy of intraoperative complete tumor resection in the experimental group was 93.33%, significantly higher than that in the control group (73.54%), with a statistical significance (P<0.05). The incidence of postoperative complications was 3.33% in the experimental group and 21.74% in the control group, with a statistically significant difference (P<0.05). Postoperative follow-up showed that good recovery rate of spinal cord function in the experimental group was 56.70%, and that in the control group was 41.09%, with a statistically significant difference (P<0.05). The recurrence rate was 0 in the experimental group and 4.34% in the control group at follow-up, with no statistically significant difference (P>0.05).

**Conclusions:**

With the application of MR holographic imaging technology in the surgical treatment of spinal cord glioma, critical clinical value can be realized. Specifically, the resection degree of spinal cord glioma can be displayed in real time, accurately, and three-dimensionally, the effect of surgical resection can be improved, surgical complications can be diminished, and the recovery of spinal cord function can be accelerated.

## INTRODUCTION

Spinal cord glioma is a relatively rare clinical disease of the central nervous system, accounting for 2%-4% of all central nervous system tumors.^1^ It can be divided into ependymoma and astrocytoma according to its cell origin. Clinically, neurosurgery is generally applied to treat spinal cord glioma. However, due to the limited transverse diameter of the spinal cord and its important physiological function, great difficulties have been caused to the treatment of spinal cord glioma. The treatment principle of spinal cord tumor is to completely remove the tumor tissues while preserving the structural and functional integrity of the surrounding tissues to the maximum extent.^2^ Preoperative systematic and detailed imaging examination plays an important role in formulating surgical plans and reducing intraoperative risks as well as postoperative complications. CT and MRI are the necessary means of examination during surgical resection of a spinal cord tumor at the current stage. Specifically, CT boasts a detailed display of bony anatomy, while MRI has a distinct advantage over CT in showing soft tissue structures, especially the tumor and its edema zone. However, the information provided by this type of image data is limited to a two-dimensional plane. In addition, due to the thickness of the scanning layer, spinal cord tumors cannot be directly observed, and one-sidedness inevitably exists.^3^ In recent years, with the innovation and development of electronic information technology, as well as increasingly mature multi-modal image fusion technology and 3D reconstruction technology, especially the application of the latest augmented reality technology, the mixture of real environment and 3D virtual model has been realized, the more complete and valuable information can be obtained via holographic images, so as to realize the interaction between virtual objects and the real world.^4^

In this way, the anatomical relationship between the tumor and the surrounding tissues was intuitively understood, and a more detailed and accurate surgical plan was formulated. However, few clinical studies on MR holographic imaging technology have been carried out. Based on this, in this study, a retrospective study was conducted on patients undergoing surgical treatment of spinal cord glioma to analyze and evaluate the application value of MR holographic imaging technology.

## METHODS

The clinical data of 53 patients with primary spinal cord glioma admitted to our hospital from January 2017 to May 2020 were retrospectively studied.

### Ethical approval

The study was approved by the Institutional Ethics Committee of Baoding No.1 Central Hospital on May 21, 2020 (No. [2020]042), and written informed consent was obtained from all participants

### Inclusion criteria:

- Patients with clear central nervous system symptoms and signs.
- Patients with detailed MRI imaging data before and after surgery.
- Patients who meet the indications for neurosurgery and agree to the operation by themselves and their family members.
- Patients who were confirmed to be primary glioma by pathological tissue biopsy after neurosurgery.
- Patients with complete clinical data and follow-up data.

###  Exclusion criteria

- Patients suspected to have myelitis after preoperative examination.
- Patients who did not receive neurosurgical treatment or were pathologically confirmed as non-gliom.
- Patients with incomplete clinical data or follow-up data. Among all patients, 29 were males and 24 were females, aged 10-61 years old, with an average of (37.89±5.34) years old.

All patients underwent neurosurgery for spinal cord glioma, with general anesthesia in the prone position followed by a routine laminectomy via a posterior median approach. For patients involving multiple segments, the spinous process and the same lamina were first milled down with a milling cutter, then connected to a fixed reduction, and microexcision was finally performed.

In the control group, intraoperative Doppler ultrasound was performed with BKL202 color Doppler ultrasound diagnostic instrument and matching probes, and epidural exploration was performed to determine the direction and range of lamina bite and fully exposed the tumor, with a frequency of 3.8-10.0MHz. Subsequently, tumor sites, boundaries, depth from the surface, and blood flow inside and outside the tumor were examined on the spinal cord surface. The spinal cord was sharply cut longitudinally in the weakest and lack of blood vessels region of the spinal cord where the tumor was located, and the adhesions to the normal spinal cord were sharply separated along the tumor boundary, either completely or in pieces. Tumors with fuzzy boundaries need to be detected by ultrasound at any time during the resection process. If residual is indicated, further excision is required.

Patients in the experimental group received MR holographic imaging technology intraoperatively, including 3D-slice software system, MR glasses, star map imaging system, laptop computer, etc. Specific operating procedures: Firstly, the original image data of the spinal cord tumor was obtained via a 3.0T MRI scanner, and then a three-dimensional CT scan was performed. Before the scan, 3-6 markers should be affixed near the patient’s surgical area, and then a three-dimensional CT thin-slice scan of the spine was performed to obtain the original image data including the markers; Subsequently, blood vessel CTA scan was performed to obtain the original image data. The above raw data were imported in DICOM format, and the software system was utilized to register the unified coordinate system for CT, MRI or CTA of the same patient. Finally, the following procedures were followed:

- The CTA data of the spine was loaded, the arteriovenous data were displayed in 3D, and the model was exported and saved in STL format through Segmentation.^5^
- The CT data of the spine was loaded, and the 3D spine model was displayed in the Segment Editor module. Skin and marker models were obtained by the same method. The skull, skin, and marker models were exported and saved.
- The MRI/CT/CTA data of the spine was loaded, and the Segment Editor module was run to display the spinal tissue in 3D, and the model was exported and saved.
- The MRI data of the tumor was loaded, the Segment Editor module was run to display the tumor in 3D, and the tumor model was exported and saved.
- The MRA/MRV data of the arteries and veins were loaded, the Segment Editor module was run to display the arteries and veins in 3D, and the model was exported and saved.
- All models were imported into the star map imaging system. Different colors and transparency of the models were set by the system, and then the holographic images containing the above models were obtained through visual rendering by the HoloLens display device

Experienced neurosurgeons wore HoloLens^6^ and carried out viewpoint tracking, rotation, translation and other operations on the HoloLens holographic image model through a laptop computer. The model was hidden and the transparency of the model was changed according to the operation requirements, so as to observe the anatomical relationship between the tumor and the surrounding blood vessels, nerves and other tissue structures. In addition, the optimal surgical procedure was determined based on the personalized holographic image of the patient. For superficial tumors, preoperative registration and fusion were performed between the holographic image and the patient’s spine in situ according to the body surface markers. Combined with the guidance of holographic images, the best placement position, flap incision design, bone flap drilling location, the best method of tumor exposure, the largest range of tumor resection, and possible intraoperative risk factors were determined, and the tumor focus was removed according to the surgical plan.

### Observation indicators

Postoperatively, MRI was utilized to review the complete resection of the tumor, and the rate of complete resection of the tumor and the incidence of postoperative complications were determined. Enhanced MRI results were used as the standard to master the accuracy of intraoperative complete tumor resection.^7^ All patients were followed up for 12 months postoperatively and were assessed for recurrence. MMS grading was used to evaluate the recovery of spinal cord physiological function postoperatively. If the preoperative grade is Grade II-V and the postoperative improvement is ≥ 1 grade, or if the preoperative grade is Grade I and the postoperative change is not observed, the recovery is rated as good. If the preoperative grade is Grade I-IV and the postoperative decrease is ≥ 1 grade, or if the preoperative grade is Grade II-V and the postoperative change is not observed, the recovery is assessed as poor.^8^

### Statistical Analysis

All data were processed by SPSS22.0 software. Enumeration data were expressed as (%) and tested by x². Measurement data presented normal distribution was expressed as (*x̅*±*s*), and t test was adopted for intra-group pairing. P<0.05 indicates a statistically significant difference.

## RESULTS

According to the analysis, there were no statistically significant differences in gender, age, MMS grade, pathological classification, tumor size and other clinical basic conditions between the two groups (P > 0.05). Table-I. Postoperative MRI reexamination showed that the tumor resection rate of the experimental group was 96.67%, which was significantly higher than that of the control group (82.61%, P < 0.05). Based on the results of enhanced MRI examination as the standard, the assessment accuracy of intraoperative complete tumor resection in the experimental group was 93.33%, significantly higher than that in the control group (73.54%), with a statistical significance (P < 0.05). Table-II.

**Table-I:** Comparison of basic clinical conditions between the two groups.

Item		Experimental group (n=30)	Control group (n=23)	X^2^/t	P value
Gender (n, %)	Male	16 (53.33)	13 (56.52)	0.442	0.064
	Female	14 (46.67)	10 (43.48)	
Age (χ̅±s, years old)		38.54±5.56	37.63±5.62	0.184	0.104
Pathological classification (n, %)	Astrocytoma	18 (60.0)	12 (52.17)	1.098	0.171
	Glioblastoma	12 (40.0)	11 (47.83)		
MMS grade (n, %)	Grade I	10 (33.33)	11 (47.83)	0.763	0.082
	Grade II	14 (46.67)	9 (39.13)		
	Grade III	4 (13.33)	2 (8.70)		
	Grade IV	1 (3.33)	1 (4.34)		
	Grade V	1 (3.33)	0	
Tumor size (χ̅±s, cm^3^)		1.24±0.22	1.18±0.31	6.833	0.077

**Table-II:** Comparison of tumor resection effect between the two groups (n, %).

Group	Number of cases	Complete resection of the tumor	Assessment accuracy of intraoperative tumor resection
Experimental group	30	29 (96.67)	28(96.55)
Control group	23	19 (82.61)	14(73.54)
t		7.384	9.022
P-value		0.001	0.000

The incidence of postoperative complications in the experimental group was 3.33%, which was far lower than the 21.74% in the control group, with a statistically significant difference (P<0.05). Table-III.Both groups were followed up for 12 months, and the recovery rate of spinal cord function in the experimental group was 56.70%, which was significantly higher than that in the control group (41.09%). The recurrence rate was 0 in the experimental group and 4.34% in the control group at follow-up, with no statistically significant difference (P>0.05. Table-IV.

**Table-III:** Comparison of postoperative complications between the two groups (n, %).

Group	Number of cases	Incision infection	Spinal infection	Bleeding	Sensory dysfunction	Number of occurrences
Experimental group	30	1 (3.33)	0	0	0	1 (3.33)
Control group	23	3 (8.70)	1 (4.34)	1 (43.44)	0	7 (21.74)
t						10.293
P value						0.001

**Table-IV:** Postoperative spinal function recovery and recurrence of the two groups (n, %).

Group	Number of cases	Good recovery of spinal cord function	Recurrence at last follow-up
Experimental group	30	17 (56.70)	0
Control group	23	11 (47.83)	1 (4.34)
X^2^		1.074	0.185
P value		0.144	0.066

## DISCUSSION

Currently, microsurgery is an important method for the treatment of tumors in the spinal cord, and preoperative and intraoperative imaging monitoring can greatly improve the accuracy and effectiveness of surgery. However, how to accurately and objectively locate the tumor during surgery, especially the tumor with fuzzy boundary, located on the ventral side of the spinal cord and accompanied by cystic degeneration and cavitation, still faces great clinical difficulties.^9^,^10^ In view of the complex anatomical structure surrounding spinal cord glioma, the imaging information provided CT, MRI, etc. has its own advantages, but these information are two-dimensional images, which cannot meet the needs of clinical surgery. In clinical surgery, 3D visualization holographic images that can intuitively and comprehensively display tumors, peripheral blood vessels, nerves and important anatomical structures are in urgent need of exploration, which can be applied to preoperative evaluation and surgical plan formulation, so as to prevent and reduce perioperative complications, and provide reliable, intuitive and comprehensive imaging information for smooth surgery.^11^

MR technology, as a new type of digital holographic imaging technology, can generate a new visual environment via virtual objects introduced in the real environment, so as to realize the coexistence of virtual and real objects as well as the real-time interaction between humans and virtual and real objects. The use of MR holographic technology as an adjuvant to neurosurgical treatment has attracted increasing clinical attention.^12^,^13^ With MR technology, not only can 2D image data be transformed into 3D stereoscopic model, but also the condition of the patient’s tumor and surrounding structures can be displayed in a real, clear, and intuitive holographic display via the reconstructed model, which is of great help to anatomy-based surgery.^8^,^14^ Skin, arteriovenous, vertebral tissues, tumors and markers displayed by MR holography can be displayed in different models according to clinical needs, and can also be subjected to transparent processing to clarify the anatomical structure between different models so as to better display tumors.^15^-^17^ MR holographic imaging technology can provide a basis for preoperative planning and intraoperative guidance.^8^ In traditional neurosurgery, clinicians make skin incisions on the patient’s scalp surface based on preoperative CT or MRI image data, body surface markers of the spine, measurement and conversion methods, and their own surgical experience. However, in order to reduce positioning error and maximize the range of tumor resection, the incision is usually made larger, and care must be taken during the operation so as to avoid important vascular and nerve injury during surgery.^18^-^21^

In this study, 53 patients with spinal glioma underwent surgery were retrospectively studied. Patients in the experimental group received MR holographic imaging technology, while those in the control group received conventional ultrasound. According to the results, the total tumor resection rate in the experimental group was 96.67%, and that in the control group was 82.61%, showing a statistically significant difference (P < 0.05). Based on enhanced MRI examination as the standard, the assessment accuracy of intraoperative complete tumor resection in the experimental group is 93.33%, significantly higher than that in the control group (73.54%), with a statistical significance (P < 0.05). The incidence of postoperative complications is 3.33% in the experimental group and 21.74% in the control group, with a statistically significant difference (P < 0.05). Postoperative follow-up showed that good recovery rate of spinal cord function in the experimental group is 56.70%, and that in the control group is 41.09%, with a statistically significant difference (P < 0.05). The recurrence rate is 0 in the experimental group and 4.34% in the control group at follow-up, with no statistically significant difference (P > 0.05). The above results are basically consistent with those reported by relevant studies.^22^,^23^

### Limitations of this study

This study was a retrospective descriptive study, with limited clinical data available and limited persuasive conclusions. Further intervention trials are needed in the future to confirm these results.

## CONCLUSION

With the application of MR holographic imaging technology in the surgical treatment of spinal cord glioma is worthy of clinical recommendation. With MR technology, the resection degree of spinal cord glioma can be displayed in real time, accurately, and three-dimensionally, the effect of surgical resection can be improved, surgical complications can be diminished, and the recovery of spinal cord function can be accelerated.

### Authors’ Contributions:

**ZL &** **JL:** Designed this study, prepared this manuscript, are responsible, accountable for the accuracy and integrity of the work.

**XL:** Collected and analyzed clinical data.

**JL:** Data analysis, significantly revised this manuscript.
